# Climatic and vegetational drivers of insect beta diversity at the continental scale

**DOI:** 10.1002/ece3.5795

**Published:** 2019-12-11

**Authors:** Douglas Chesters, Philip Beckschäfer, Michael C. Orr, Sarah J. Adamowicz, Kwok‐Pan Chun, Chao‐Dong Zhu

**Affiliations:** ^1^ Key Laboratory of Zoological Systematics and Evolution Institute of Zoology Chinese Academy of Sciences Beijing China; ^2^ Forest Inventory and Remote Sensing Faculty of Forest Sciences and Forest Ecology Göttingen University Göttingen Germany; ^3^ Northwest German Forest Research Institute Göttingen Germany; ^4^ Department of Integrative Biology & Biodiversity Institute of Ontario University of Guelph Guelph ON Canada; ^5^ Department of Geography Hong Kong Baptist University Kowloon Tong Hong Kong S. A. R. China; ^6^ University of Chinese Academy of Sciences Beijing China

**Keywords:** beta diversity, climate, insects, remote sensing

## Abstract

**Aim:**

We construct a framework for mapping pattern and drivers of insect diversity at the continental scale and use it to test whether and which environmental gradients drive insect beta diversity.

**Location:**

Global; North and Central America; Western Europe.

**Time period:**

21st century.

**Major taxa studied:**

Insects.

**Methods:**

An informatics system was developed to integrate terrestrial data on insects with environmental parameters. We mined repositories of data for distribution, climatic data were retrieved (WorldClim), and vegetation parameters inferred from remote sensing analysis (MODIS Vegetation Continuous Fields). Beta diversity between sites was calculated and then modeled with two methods, Mantel test with multiple regression and generalized dissimilarity modeling.

**Results:**

Geographic distance was the main driver of insect beta diversity. Independent of geographic distance, bioclimate variables explained more variance in dissimilarity than vegetation variables, although the particular variables found to be significant were more consistent in the latter, particularly, tree cover. Tree cover gradients drove compositional dissimilarity at denser coverages, in both continental case studies. For climate, gradients in temperature parameters were significant in driving beta diversity more so than gradients in precipitation parameters.

**Main conclusions:**

Although environmental gradients drive insect beta diversity independently of geography, the relative contribution of different climatic and vegetational parameters is not expected to be consistent in different study systems. With further incorporation of additional temporal information and variables, this approach will enable the development of a predictive framework for conserving insect biodiversity at the global scale.

## Graphical abstract

An informatics system was developed to integrate terrestrial data on insects with environmental parameters and to infer biogeographic maps. Beta diversity was driven by geographic, climatic, and vegetation gradients.

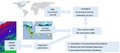

## 1 INTRODUCTION

The primary foundation on which conservation planning is built is the distribution of beta diversity (Buckley & Jetz, 2008; Margules & Pressey, 2000). Beta diversity is the difference in community composition at two or more sites, capturing the spatial dimension of biodiversity turnover, and is effective in identifying factors responsible for community assembly (McGill, Enquist, Weiher, & Westoby, 2006). There are thought to be three mechanisms which result in differences in composition between sites and thus account for much of beta diversity; the match between environmental conditions and organismal requirements, the dispersal abilities of the organism and the physical characteristics of the environment (Nekola & White, 1999), and interactions between co‐occurring species (Cornell & Lawton, 1992). It is necessary to gain some understanding of the relative contribution of these candidate drivers of beta diversity in order to make accurate predictions, for example, environmental filtering may result in very similar community structure at two sites even if they are disparate and geographically isolated (Penone et al., 2016). There are indices of beta diversity using species composition (Koleff, Gaston, & Lennon, 2003), function (Petchey & Gaston, 2002), and those derived from phylogeny (Webb, Ackerly, McPeek, & Donoghue, 2002). In some cases, these null models are insufficient to account for diversity gradients, and rarefaction of sites to a higher‐level administrative area or geospatial grid is necessary (Sandel, 2018).

Our knowledge on global patterns in beta diversity of insects remains incipient as they are notoriously laborious to survey. However, insect composition may be predicted in areas which have not been sampled through co‐opting surrogate information, particularly environmental data, which is usually geographically comprehensive (Ferrier, 2002). Earlier methods for modeling beta diversity were built on single‐species approaches but are considered of minimal utility in cases where sampling is particularly sparse (Ferrier & Guisan, 2006). In addition, classical regression can be problematic when comparing (geographic and environmental) distance matrices due to pseudoreplication (not to be confused with spatial autocorrelation of composition, or distance decay). Two approaches which address such issues when modeling dissimilarity as a function of geography and the environment are the multiple regression on distance matrices (MRM) and generalized dissimilarity modeling (GDM; Legendre, Borcard, & Peres‐Neto, 2005; Tuomisto & Ruokolainen, 2006). MRM is built on regression and the Mantel test and compares the dissimilarity matrix of the response data to one or more matrices of explanatory variables, which include geographic distance and environmental variables (Lichstein, 2007). By contrast, GDMs uses nonlinear (I‐spline) functions, which appear to be a better fit for typical compositional and environmental gradients than linear functions (Fitzpatrick et al., 2013) and have a wide range of applications (Ferrier, Manion, Elith, & Richardson, 2007).

Previous work on environmental drivers of insect beta diversity has typically considered climatic and habitat parameters. Being ectotherms (with exceptions such as bumblebees), insects can be particularly constrained by climatic conditions (Speight, Hunter, & Watt, 2008). For example, the stringency of temperature constraint for Chironomidae (nonbiting midges) development is such that they are used for inferring past environments (Eggermont & Heiri, 2012). Climate dictates insect distribution not only directly through physiological limits due to temperature, precipitation, and humidity, but also via vegetation type and abundance, and through multitudinous species interactions (Stange & Ayres, 2010).

Given the sheer number of insect species and habitats they occupy, the degree to which insects follow generalized ecological principles remains unclear, and specific tests of the relative contributions of geography, climate, and land type to insect beta diversity are sparse, although some work has been conducted in widely studied insects such as bees and butterflies. In the interface of agricultural and seminatural sites in Israel, a beta diversity shift attributed to species richness gradient was observed in butterflies, the strength of which was influenced by the level of precipitation (Pe'er, van Maanen, Turbé, Matsinos, & Kark, 2011). For the bees of Western Canada, there was a considerable alignment of assemblages to climatic and habitat classes, probably due to conserved traits that permit existence in certain areas (Villalobos & Vamosi, 2018). In Nordic ground and diving beetles, beta diversity was most strongly correlated with gradients in geographic distance, annual temperature, and open land (Heino, Alahuhta, Fattorini, & Schmera, 2018). Krasnov et al. (2015) compared environmental and host factors on composition and trait selection in Palearctic fleas. However, there is as yet no attempt at a continental‐scale comparison of climatic and land‐use drivers for insects as a whole, which is a major omission considering their unparalleled diversity and integrality to the ecosystem.

Herein, our general goal is the initiation of continental‐scale beta diversity maps for insects. Our specific questions are (a) do environmental gradients of climate and vegetation drive dissimilarity in insect composition independently of geographic distance and (b) if so, are there specific parameters which contribute most to compositional shift. Earth observation and climate data are prime candidates for global environmental drivers of insect distribution (Bush et al., 2017); hence, our focus herein is bioclimate (Fick & Hijmans, 2017) and vegetation indices derived from remote sensing (Townshend et al., 2011). This foundational system on contemporary insect beta diversity paves the way for a temporally predictive framework.

## 2 METHODS

**Figure 1:**
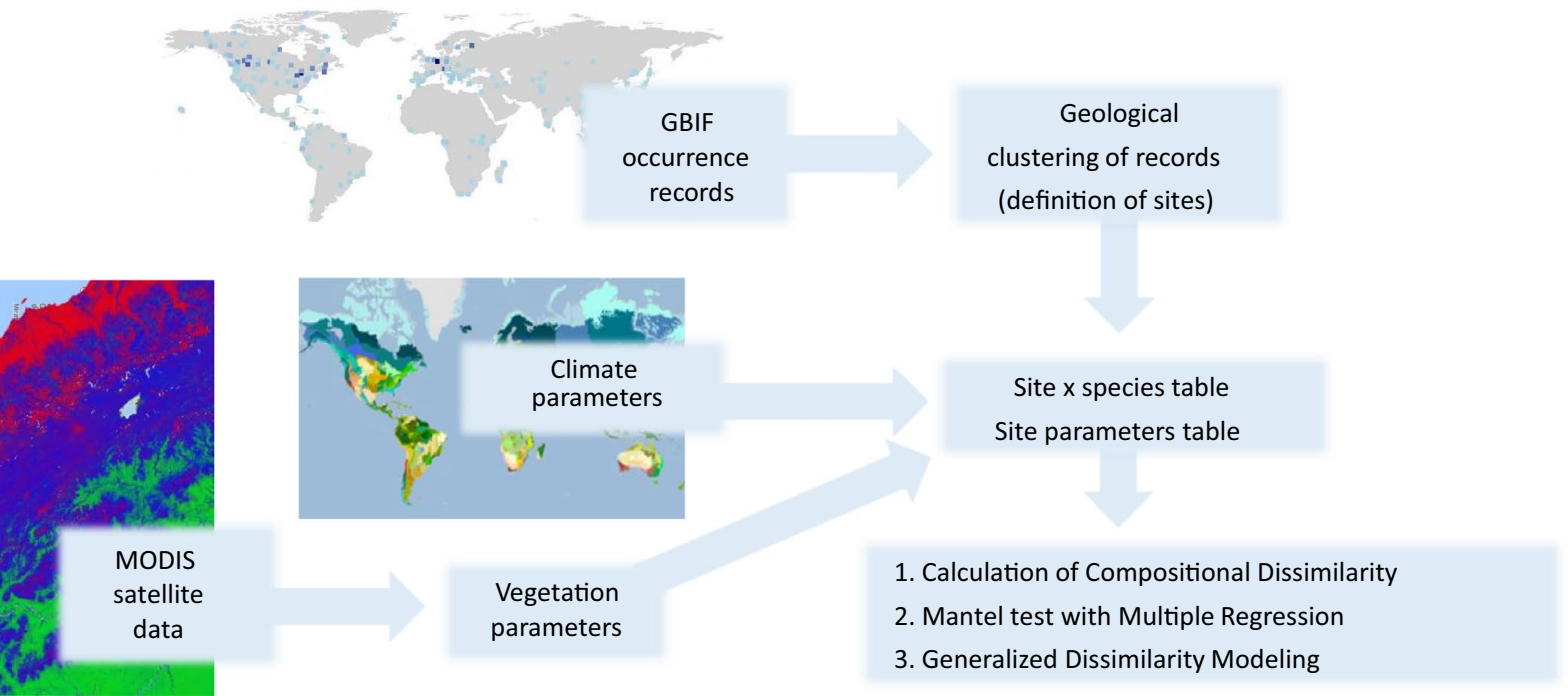
Flowchart giving main steps in the mining, processing, and analysis framework herein

An overview of the analysis pipeline herein is given in Figure 1.

### 2.1 Distribution

Contemporary insect distributions were inferred from observation records. We conducted three case studies: a global model and two continental‐scale models for the most comprehensively sampled regions of North and Central America and Western Europe. For the new world, Central America was included as to encompass the well‐sampled tropical regions of Costa Rica and Panama. Geographic occurrence records were retrieved from the Global Biodiversity Information Facility (GBIF), an international network and research infrastructure which provides open access to data about species occurrences on earth. GBIF records for all insects were downloaded (GBIF Occurrence Download https://doi.org/10.15468/dl.hciamo accessed via http://GBIF.org). Coordinates were grouped into sets of geographic samples. Euclidean distances between coordinates were calculated using the “ecodist” R package (Goslee & Urban, 2007), and then distances were clustered with mcl (van Dongen, 2000). The ‐I and ‐pi options were adjusted to reduce formation of large regions which overlap multiple ecoregions. Remaining observations which occurred at boundaries (coordinates at one decimal place, roughly corresponding to 5 km) between ecoregions were removed.

### 2.2 Environmental variables

Based on geographic coordinates, occurrences were classified for climate (Kottek, Grieser, Beck, Rudolf, & Rubel, 2006) and ecoregion (Olson et al., 2001). Currently, Kottek et al. (2006) is the most popular system to define Köppen–Geiger climate type based on explicit rules of temperature and precipitation conditions. Köppen–Geiger climate zones correspond to partitions in composition in some insects (Brugger & Rubel, 2013), although to date studies of climate classes and higher taxonomic insect groups are rare. We also used the WWF's “Terrestrial Ecoregions of the World” classification (available at http://maps.tnc.org/gis_data.html; Note, “realm” and “biome” are broader classes within the same system, although not applied here). Ecoregions contain biotic and abiotic features which promote specific communities (e.g., Olson et al., 2001); thus, patterns in distribution might delineate with physical factors, such as hydroclimatic conditions, or functional considerations such as nutrient cycles (Cox, Moore, & Ladle, 2016). Ecoregions have been used extensively for looking at global habitat loss and conservation assessment (Hoekstra, Boucher, Ricketts, & Roberts, 2005). Other bioregion classifications exist, although are either largely derived from the terrestrial ecoregions (The Nature Conservancy, 1997; http://maps.tnc.org/gis_data.html) or are regionally restricted (EPA ecoregions, a 3‐level system for US, Canada, Mexico; and the Global 200).

**Figure 2:**
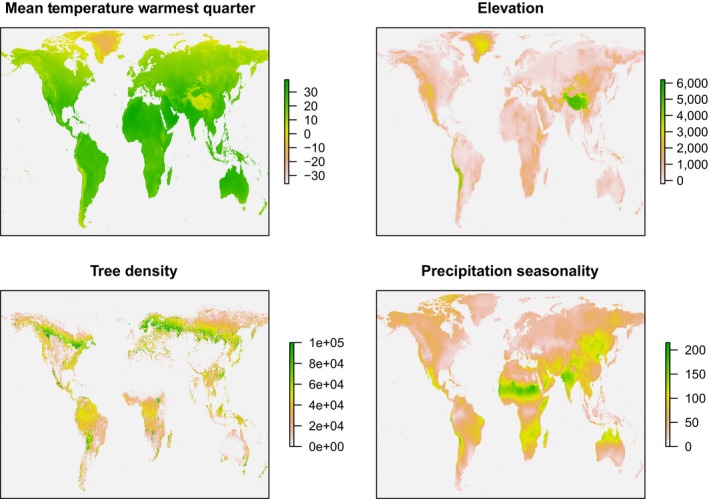
Four mapped example variables which are candidate drivers of insect beta diversity

In addition to classifications, we retrieved candidate environmental data under the criteria that they (a) are likely to constrain the distribution of insects, (b) are available for all terrestrial regions, and (c) are likely to be produced regularly (for the purpose of further development of a temporal dimension). Figure 2 shows four examples out of the 23 variables retrieved. The 19 bioclimate variables of WorldClim version 2 (Fick & Hijmans, 2017) were obtained from http://worldclim.org/version2, at 5 min resolution and in GeoTIFF format. For each sample site, climate parameters were extracted from the geotiff file using the gdallocationinfo function of the Geospatial Data Abstraction Library (GDAL/OGR Contributors, 2018). Next, estimates of vegetation cover were derived from the Moderate‐Resolution Imaging Spectroradiometer (MODIS) Vegetation Continuous Fields (VCF) product (MOD44B V051, Townshend et al., 2011). The VCF product (Figure S1.1) provides annual global estimates of vegetation cover in terms of tree vegetation, herbaceous vegetation, and bare ground percentages. It is generated using monthly composites of Terra MODIS 250 and 500 m Land Surface Reflectance data, including all seven spectral bands and land surface temperature. To reduce interannual fluctuations caused by atmospheric noise, median values of the three cover categories were calculated over the 3‐year period 2013–2015 for the nominal year 2014. Values were extracted at a 250 m pixel scale. For a small portion of sampling locations, no values were available at this scale; thus, at these locations values were extracted from the nearest neighbor pixel that contained data. To facilitate this, the VCF product was resampled (nearest neighbor resampling) at steps of 250 m until a pixel size of 2,000 m was reached. All image processing and sampling was done using Google Earth Engine (Gorelick et al., 2017).

### 2.3 Data analysis

Analysis was conducted in R, using the libraries “picante” (Kembel et al., 2010), “vegan” (Dixon, 2003), “ecodist” (Goslee & Urban, 2007), and “gdm” (Manion, Lisk, Ferrier, Nieto‐Lugilde, & Fitzpatrick, 2019), although note the size of the datasets precluded some functions. For compositional beta diversity, pairwise dissimilarity was calculated according to a variant of the Morisita measure, which is based on the probability that two individuals from each of site pair are of same species (Magurran, 2004). Although the Bray–Curtis measure is perhaps the mostly commonly used in community ecology, Morisita, and derived indices have proven more robust against incomplete sampling and unequal sample size (Barwell, Isaac, & Kunin, 2015; Magurran, 2004; Wolda, 1981), with the Horn–Morisita variant more flexible on input data type.

Two approaches were used to account for autocorrelation in distance matrices, MRM and GDM. MRM returns the proportion of variance in beta diversity attributable to the environmental variables. Secondly, GDM extends traditional regression of composition and spatial/environmental distances with nonlinear functions, which more appropriately fit the asymptote in composition observed over large areas (Fitzpatrick et al., 2013). Geographic distance (input as separate latitude and longitude) was modeled along with elevation, 19 bioclimate variables, and three vegetation cover variables. Significant I‐splines were extracted, plotted individually, and transformed to biological space, and predicted dissimilarities were estimated based on the model. Beta diversity measures used in GDM were species composition based on Horn–Morisita. Species richness was used for site weighting in GDM, which reduces bias when using ad hoc presence data (Ferrier et al., 2007). Finally, the spatial pattern in insect diversity was mapped for the whole area of interest by predicting beta diversity between sites using the fitted model with significant environmental variables (Manion et al., 2019). For visualizing the spatial pattern, dimensionality was reduced with a principal component analysis (PCA), and the first three components used in plotting.

## 3 RESULTS

### 3.1 Case study 1, Global

**Figure 3:**
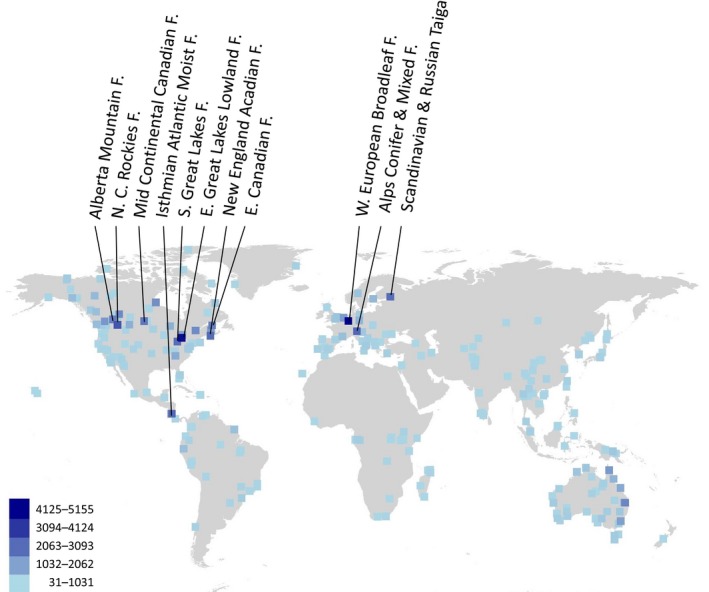
Insect species per ecoregion as analyzed herein. Points are positioned at most frequently sampled area within ecoregion. Most thoroughly sampled ecoregions are labeled. Abbreviations: C, central; F, forests; N, north; S, south; W, west

A total of 21,542,045 records were parsed with geographic coordinates (Figure 3). Ignoring 264,256 species observed at <30 sites and sites with <300 observations (thresholds selected to meet computational demands), a matrix of 8,553 sites and 38,388 species was analyzed. About 67.8% of null deviance was explained by the model, with geographic distance the main driver by magnitude. Independent of geographic distance, gradients in six environmental variables were significant in driving insect compositional shifts; precipitation of the driest quarter (magnitude 0.7), tree density (magnitude 0.4), elevation (0.4), precipitation seasonality (0.15), temperature of the driest quarter (0.1), and diurnal range (0.1). Based on the model, the biogeography of insects was predicted for all terrestrial regions (Figure S1.2).

### 3.2 Case study 2, North and Central America

For North and Central America, there were 304,090 GBIF records subject to climatic and land‐type classification, with filtered tables of dimensions 931 sites and 5,117 species. Community dissimilarity was greater between sample sites of different ecoregions (*r* = .7025, *p* < .001) or climes (*r* = .3177, *p* < .001) than between sites of the same class. According to MRM, a model including geographic, climate, and vegetation parameters could explain a reasonable degree (*r* = .344, *p* < .001, Table 1) of variance in compositional dissimilarity, although the contribution of individual parameters was negligible. The fit was greater with inclusion of the 19 climate parameters (*r* = .337) than inclusion of the three vegetation parameters (*r* = .252).

**Table 1:** *r* ^2^ values of MRM tests

Model components	N. & C. America	W. Europe
Compositional dissimilarity ~ geographic	.237	.094
Compositional dissimilarity ~ geographic + climate	.337	.191
Compositional dissimilarity ~ geographic + vegetation	.252	.108
Compositional dissimilarity ~ geographic + climate + vegetation	.344	.198

Note

*p* < .001 in all cases.

**Figure 4:**
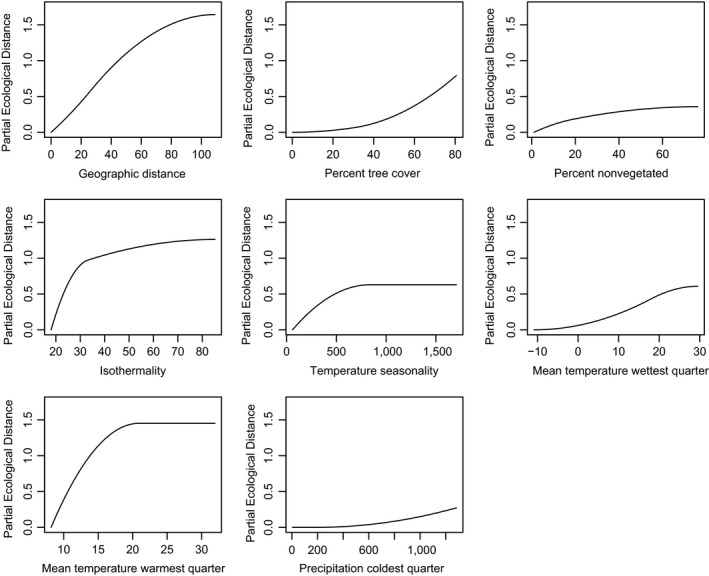
For N. & C. America, I‐splines for significant individual geographic or environmental variables from the fitted generalized dissimilarity modeling. *Y*‐axis shows the partial ecological distance, that is, the magnitude of compositional change according to the environmental gradient

**Figure 5:**
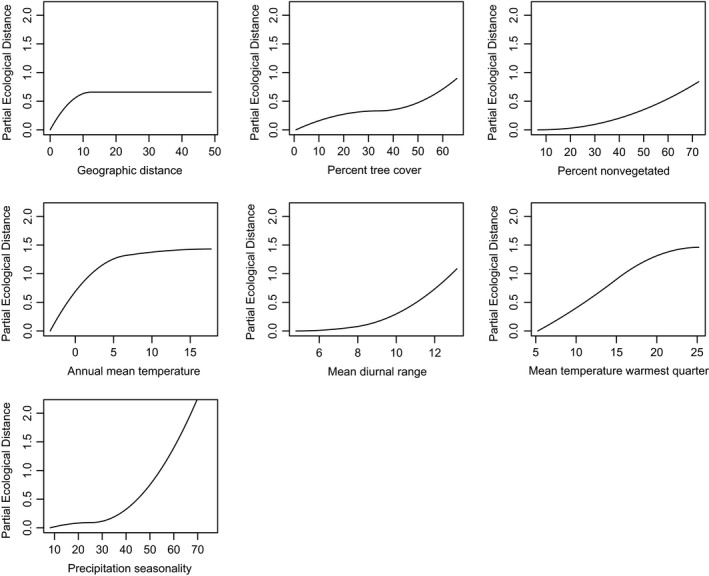
For W. Europe, I‐splines for significant individual geographic or environmental variables. *Y*‐axis shows the partial ecological distance

**Figure 6:**
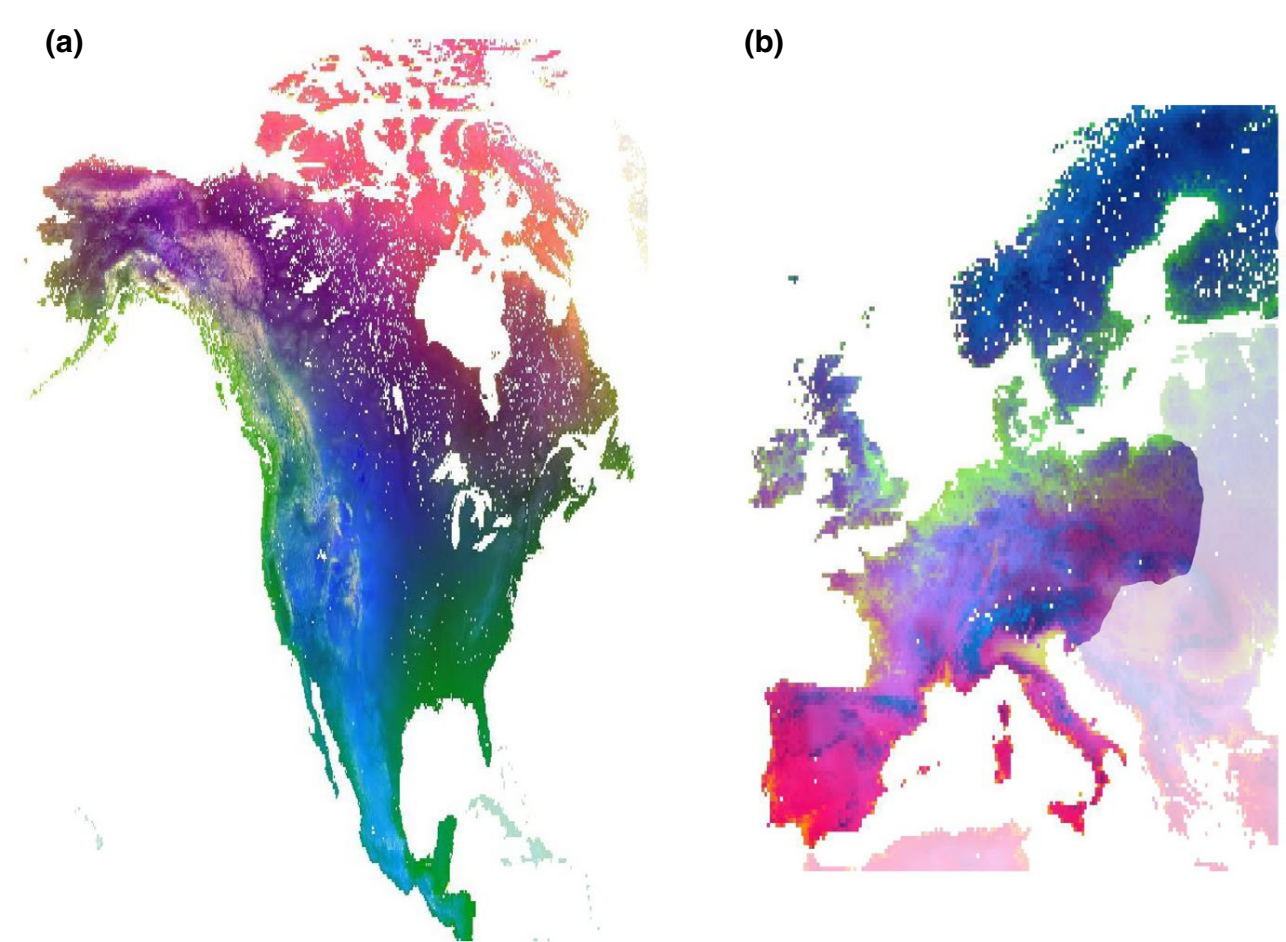
Continent‐specific insect beta diversity for (a) N. & C. America and (b) W. Europe. Based on the generalized dissimilarity modeling, predicted beta diversity between points was transformed to biological space on significant environmental rasters. The space was depicted through the first three axes of a principal component analysis assigned to the colors red, green, and blue. Areas not included in the model are shaded

The I‐spline functions of the GDM have been favored recently as a better fit of the nonlinear nature of composition and environmental gradients, which are particularly relevant at broad scales. Accounting for geographic distance and including all climatic and vegetation parameters, 56% of null deviance was explained by the model. The fitted splines for all environmental variables significant in both GDM and MRM are shown in Figure 4 (and for W. Europe in Figure 5), with individual variables transformed to biological space in Figure S1.3, and predicted beta diversity across the continents in Figure 6. The greatest degree of compositional change occurred along the geographic distance gradient. Otherwise, temperature‐based climatic parameters featured more heavily than precipitation or land‐type parameters. The curves for RS‐derived vegetation indices were complementary, with the greatest degree of compositional change occurring in denser vegetation coverages.

### 3.3 Case study 3, West Europe

A total of 2,997,351 GBIF records were obtained for which climatic and land‐type classifications could be made, with 4,085 sites and 8,523 species retained after filtering steps. Although less pronounced than for N. & C. America, community dissimilarity in W. Europe was also greater between samples of different ecoregion (*r* = .2855, *p* = .001) or climes (*r* = .1816, *p* = .001) than within. The MRM model of geographic, climate, and vegetation explained 0.198 of variance in composition (Table 1). As previously, the model improved more so from the inclusion of climatic rather than vegetation parameters. In the GDM, 29% of null deviance was explained, with significant fitted splines shown in Figure 5 and biological space mapped in Figure S1.4. In W. Europe, geographic distance was not the most prominent correlate of compositional variance and only showed an influence at short ranges. As observed in N. & C. America, change in tree cover at the denser ends of the scale corresponded to composition changes, and gradients in temperature‐based climatic variables were key predictors of compositional changes, particularly mean summer temperature.

Naturally, the dataset includes many species in which the distribution is insufficiently represented. Limiting the analysis to 362 species which were very highly sampled (>1,000 observations), results were very similar, with 27% of variance explained, and similar magnitudes for environmental parameters. Similarly, repeating the analysis only on observations of Lepidoptera species, being data‐rich while ecologically homogeneous in comparison to other insect groups, we find a reduced model fit (23.8% of null deviance explained) but remarkably similar magnitude and pattern in environmental drivers (Figure S1.5). These findings suggest the analysis is unaffected by species with poor distribution information, although we cannot discount that the results reflect more so the environmental needs of those species well represented in the database.

## 4 DISCUSSION

### 4.1 Environmental drivers of insect diversity

Our initial analyses used established environmental classifications of Köppen–Geiger climatic and WWF ecoregion. In partitioning insect diversity across climes and ecoregions, it was found that forests harbored the overwhelming majority of compositional variation in both continents, likely related to their considerable land area. We also found modest unique composition in the tundra–taiga of Canada and cold and polar climes of both continents. The tropics are generally understudied, and herein this clime was represented only by a geographically restricted region of Central America. Perhaps as a result, the tropical samples were relatively homogenous, although there have been conflicting findings on the level of turnover of tropical insects (d'Souza & Hebert, 2018; Novotny et al., 2007). Otherwise, greater diversity was correlated with decreasing latitude, increasing temperature, greater tree cover, and less open ground. However, the utility of contemporary classifications can be limited in that they are not amenable to automated updates, nor to some key modeling methods. For this reason, we conducted modeling on the underlying environmental variables, which represent a more sustainable data source for development of predictive modeling of beta diversity.

Of the 23 environmental variables assessed as candidate drivers of insect distribution, percent tree cover was one of the few which showed a consistent magnitude and pattern over the case studies and methods, with the finding that gradients of tree cover of >40% drove compositional dissimilarity of insects, independently of geographic distance. To date, tree density effects on alpha diversity have received considerably more study than beta diversity. For example, tree species diversity was found to drive alpha diversity of herbivorous insects only in densely planted forest plots and not sparse plots (Barantal et al., 2019); while reduced tree density and related abiotic effects resulting from urbanization increased herbivore insect abundance and decreased parasitism rate (Dale & Frank, 2014), indicating taxon‐specific effects. There has been little attention on tree density effects on insect beta diversity, although it is known that for cursorial insects inhabiting trees, minor reductions in vegetation density can represent considerable barriers in connectivity (Adams, Schnitzer, & Yanoviak, 2019). Candidate mechanisms responsible for tree density effects might be that there is a threshold in resource density at which insects will begin to use the resource (Verschut, Becher, Anderson, & Hambäck, 2016). Further, there is a well‐established theoretic framework for plant‐herbivore interaction, in which plant traits and interactions with higher trophic groups are mediated by plant neighborhood (Underwood, Inouye, & Hamback, 2014), while tree cover at the scale of plot or forest is well known to drive insect communities in both alpha and beta diversity, probably via habitat availability and microclimatic differences between dense and open forests (e.g., Friess et al., 2019; Penone et al., 2019).

Of the climatic variables, mean temperature of the warmest quarter most consistently drove compositional shifts, with shifts occurring in the range 10–20°C. Again, to date studies specifically comparing bioclimate and vegetation drivers on insect beta diversity have been sparse. Climate was not dominant in structuring composition in the case of fungus‐associated arthropods in beech forest (Friess et al., 2019). In other study systems, mean summer temperature was found to be the primary climatic variable driving alpine plant diversity (Baldwin‐Corriveau, 2012) and beta diversity of grassland and savanna plots in South Africa (Jewitt, Goodman, O'Connor, Erasmus, & Witkowski, 2016), and compositional and phylogenetic beta diversity of snakes in the Atlantic forest hotspot of South America (Moura, Costa, Argôlo, & Jetz, 2017).

### 4.2 Insect biogeography

Western Europe, particularly the north, has by far the most comprehensive data on insect distribution. Geographic patterns in insect compositions were revealed through applying the model to environmental data for the whole region (Figure 6). In the north of Scandinavia, compositions strongly followed the axis of the peninsula, with a marked boundary (Figure 6) corresponding to that between the taiga and birch‐forest ecoregions, with several environmental variables following the same axis (Figure 2, Figure S1.4). The Bothnian bay is apparent as a considerable barrier to insects; indeed, it is frozen for half of the year and known to be harsh for biota (Ojaveer et al., 2010). In contrast, compositional mixing is considerable further south in the Baltic Sea, between samples of Sarmatic mixed forests of S. Sweden and S. W. Finland, via the Åland islands. Beta diversity between Fennoscandia and central Europe correlated mostly with temperature and tree density gradients (Figure S1.4). Low beta diversity was inferred through a large swath of low‐lying coastal land from the English Channel through to the Baltic Sea, whereas high, temperature‐driven beta diversity was predicted between the Pyrenees Conifer Forests, Alps conifer, and Carpathian Montane Forests and their respective surrounding sites. Precipitation seasonality was predicted to drive beta diversity observed south toward the three Mediterranean peninsulas.

### 4.3 Future directions in insect diversity mapping

Currently, the quality of data is quite heterogeneous; occurrence data may be available only from parts of a species' distribution, different regions are sampled at different intensity (Figure 3), earth observations are used for a period other than the recording of the specimen, and spatial resolution is very coarse. Further, while the way we have developed this framework facilitates integration of a predictive temporal dimension, the inclusion of this would compound the issue of incomplete insect data. Gaps in knowledge on insect species diversity and distribution are long known (e.g., Anderson, 2012; Graham, Ferrier, Huettman, Moritz, & Peterson, 2004). While the taxonomic literature can leave the impression of detailed knowledge on distribution for most insects, these information are not well databased, georeferenced, nor centralized. Lack of distribution data might be alleviated with integration of other sources. We select GBIF as it is emerging as the primary source for species distributions and the public database with the largest number of occurrence records, but regional efforts (e.g., Atlas of Living Australia) will maximize global coverage for insect diversity mapping. Further, existing museum insect collections and records need to be digitized, and while there are no significant technological hurdles to doing this (Blagoderov, Kitching, Livermore, Simonsen, & Smith, 2012; Hebert et al., 2013; Vollmar, Macklin, & Ford, 2010), there is a pressing need for addressing misidentifications in collections. In parallel, standardized insect sampling combined with next generation sequencing will be able to obtain contemporary insect biodiversity data at high levels of throughput (Morinière et al., 2016; Yu et al., 2012), although enthusiasm for such technological solutions should be tempered, for there remains considerable challenges in obtaining historical samples and in interpreting OTU which lack reference DNA barcodes. Before solutions to improving the quality of insect data are found, it would be expected that widespread species would be overrepresented as they tend to be the first to be recorded, which can lead to underestimating beta diversity (Ruokolainen, Tuomisto, Vormisto, & Pitman, 2002). As such, the current results should be considered conservative.

For environmental information, earth observation data are perhaps the critical source that will allow predictions on land‐use changes (Bush et al., 2017). Besides development of a system of temporally matched observations and environmental variables, current development of remote sensing techniques and the analysis of historically collected satellite data might improve the ability of remote sensing products to predict the distributional patterns of insect diversity. The MODIS VCF data used in our study, for example, have the limitation that it only provide estimates of vegetation cover and does not provide information on whether trees are part of a natural forest or of a monospecific plantation. This might be problematic, for example increasing density of monocultures can show insect abundance trends which differ from patterns typically observed in natural forests, such as increased pest infestations (e.g., Al Shidi, Kumar, Al‐Khatri, Albahri, & Alaufi, 2018). Neither does it provide information on other forest characteristics, such as tree age and level of disturbance, which also influences insect community structure (Perry et al., 2016). The incorporation of land cover history derived from time series analyses (such as Landsat, MODIS or Sentinel‐2) might be beneficial in this context. The open data policy of Landsat and Sentinel potentially allows for such an approach, and recently published datasets aiming at capturing forest cover dynamics and human disturbance (Hansen et al., 2013) provide starting points to explore this approach. Further, the free, global availability of Sentinel‐1 SAR data potentially enable the assessment and incorporation of vegetation structural attributes (Schmidt et al., 2017) into large‐scale assessments and analyses of insect communities. Though there have been several promising studies that showed correlations between remote sensing products and ecosystem structure, habitat conditions, and animal communities, it must be acknowledged that we are still in an early stage of deriving reliable indicators for biodiversity information from earth observation data (Bush et al., 2017).

There are many other candidate terrestrial variables both which are likely to impact insect community assembly, and that can viably be integrated, and therefore would be likely to increase explanatory power of GDMs. Examples include distance to coast (Bivand & Rundel, 2017), human footprint indicators (Venter et al., 2016), and measures of topographic heterogeneity between sites, which often better reflect spatial isolation of sites than geographic distance alone (Panda, Behera, Roy, & Biradar, 2017; Stein et al., 2015). Other sources might be the rapidly improving global plant diversity maps (Keil & Chase, 2019), as well as pesticide use records (Gibbs, Mackey, & Currie, 2009), species traits (McGill et al., 2006), and species interactions (Brooks et al., 2012). These variables include both drivers operating on evolutionary time scales and those human‐induced, both of which might have a considerable impact on insects. Finally, current methodology in predicting beta diversity might need to adapt to current ecological thinking, which suggests that in addition to community assembly being a product of environmental conditions, it is also both driver and result of ecosystem functions such as pollination and biomass production (van der Plas, 2019). In any case, the need to improve our ability to predict how insect diversity is distributed and how it is changing could not be more pressing.

## CONFLICT OF INTEREST

None declared.

## AUTHOR CONTRIBUTIONS

D.C., K.P.C., and C.D.Z. conceived/supervised the study; D.C., K.P.C. and P.B. mined and processed data; D.C. and K.P.C. analyzed the data; and D.C. wrote the paper, with input from all coauthors.

## Data Availability

“comm” table (site * species) and site parameters table, each for the two regions of N. & C. America and W. Europe, is made available at https://doi.org/10.5061/dryad.dbrv15dwr.
