# Supplementary material for: Climatic and vegetational drivers of insect beta diversity at the continental scale
Source: Ecol Evol. 2019 Dec 11;9(24):13764–75. doi: 10.1002/ece3.5795 (PMC6953656; doi:10.1002/ece3.5795)

Figure S1.1: The MODIS Vegetation Continuous Fields (MOD44B V051) product depicts estimates of vegetation cover of Earth’s terrestrial surface, at 250 m resolution. It provides a gradation of three components: percent tree cover, percent non-tree vegetation cover, and percent bare ground. Panels (a) and (b) show composites of the three components with tree cover displayed in green, non-tree vegetation cover in blue, and bare ground in red. For the magnified area displayed in (b), the components tree cover (c), bare ground (d) and non-tree vegetation (e) are displayed individually. (Background: Google Maps’ layer (Map data ©2018 Google, INEGI).


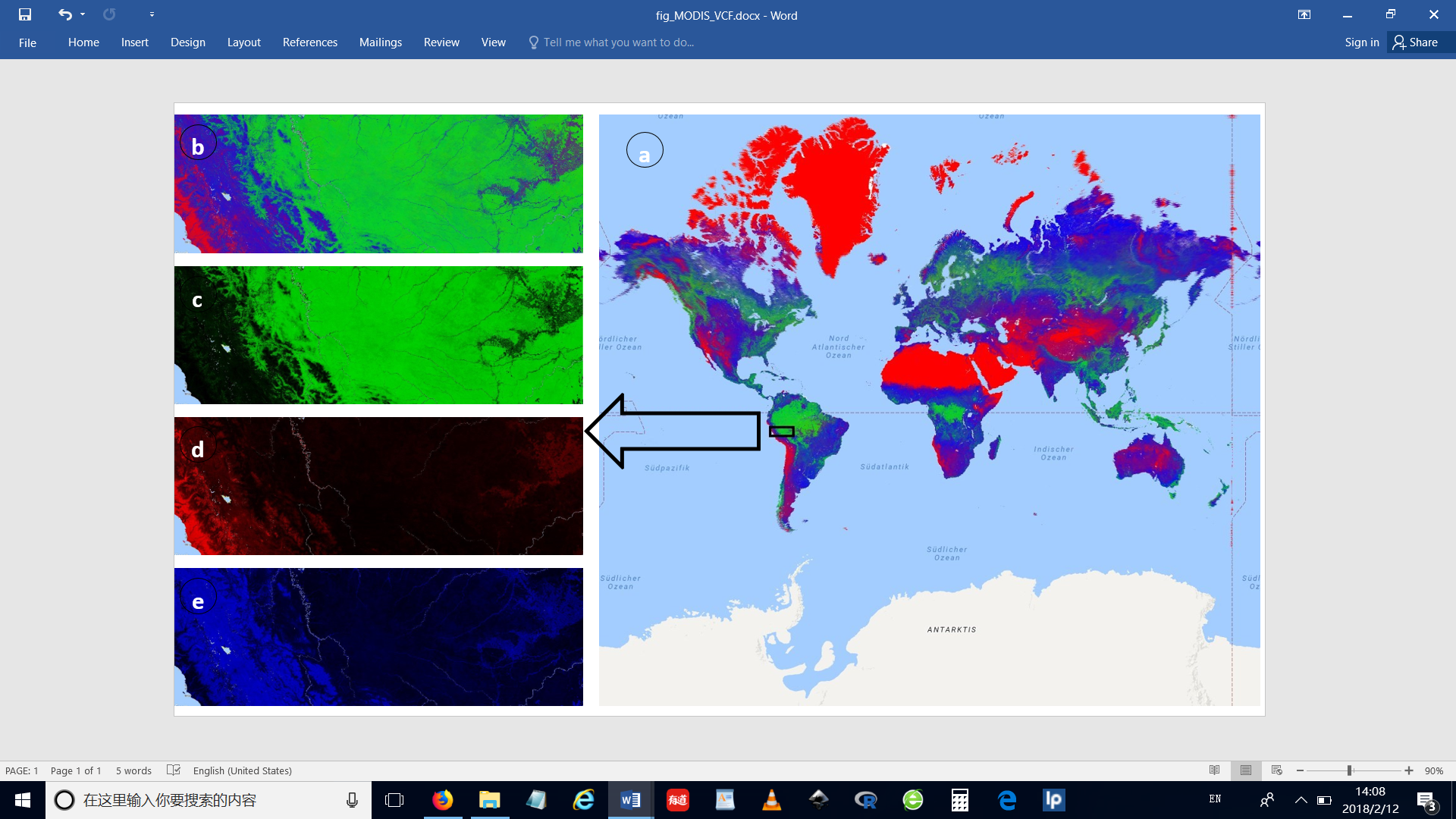


Figure S1.2: Entomobiogeographic map of the world, automatically generated through predictive dissimilarity modelling of all publically available insect distribution data on GBIF, against significant environmental variables.


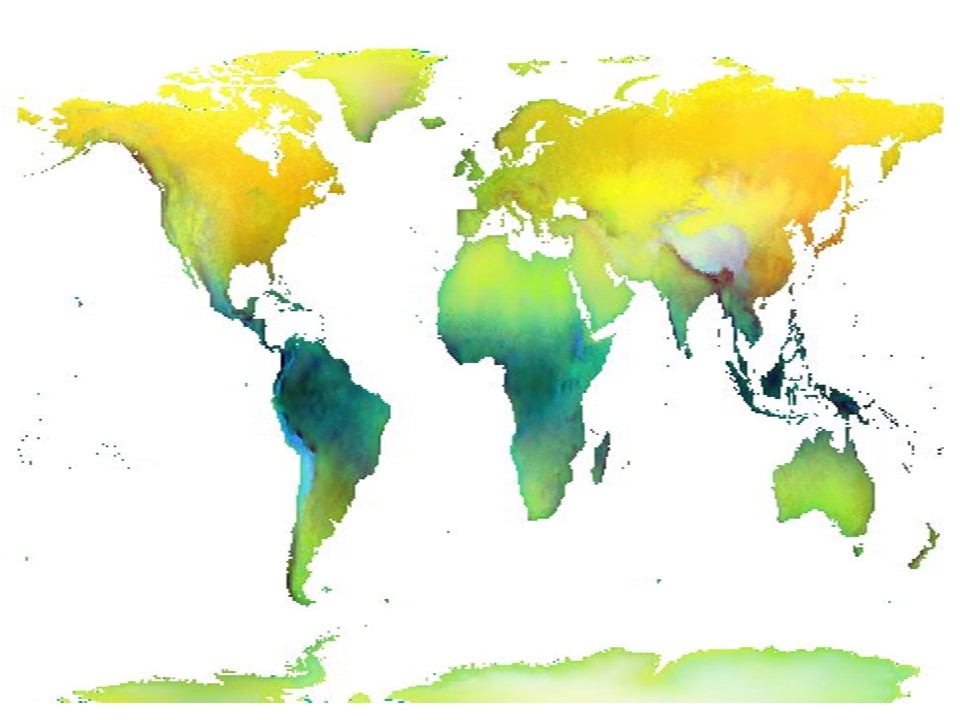


Fig S1.3: Individual environmental variables in the GDM transformed to biological space for N. & C. America.


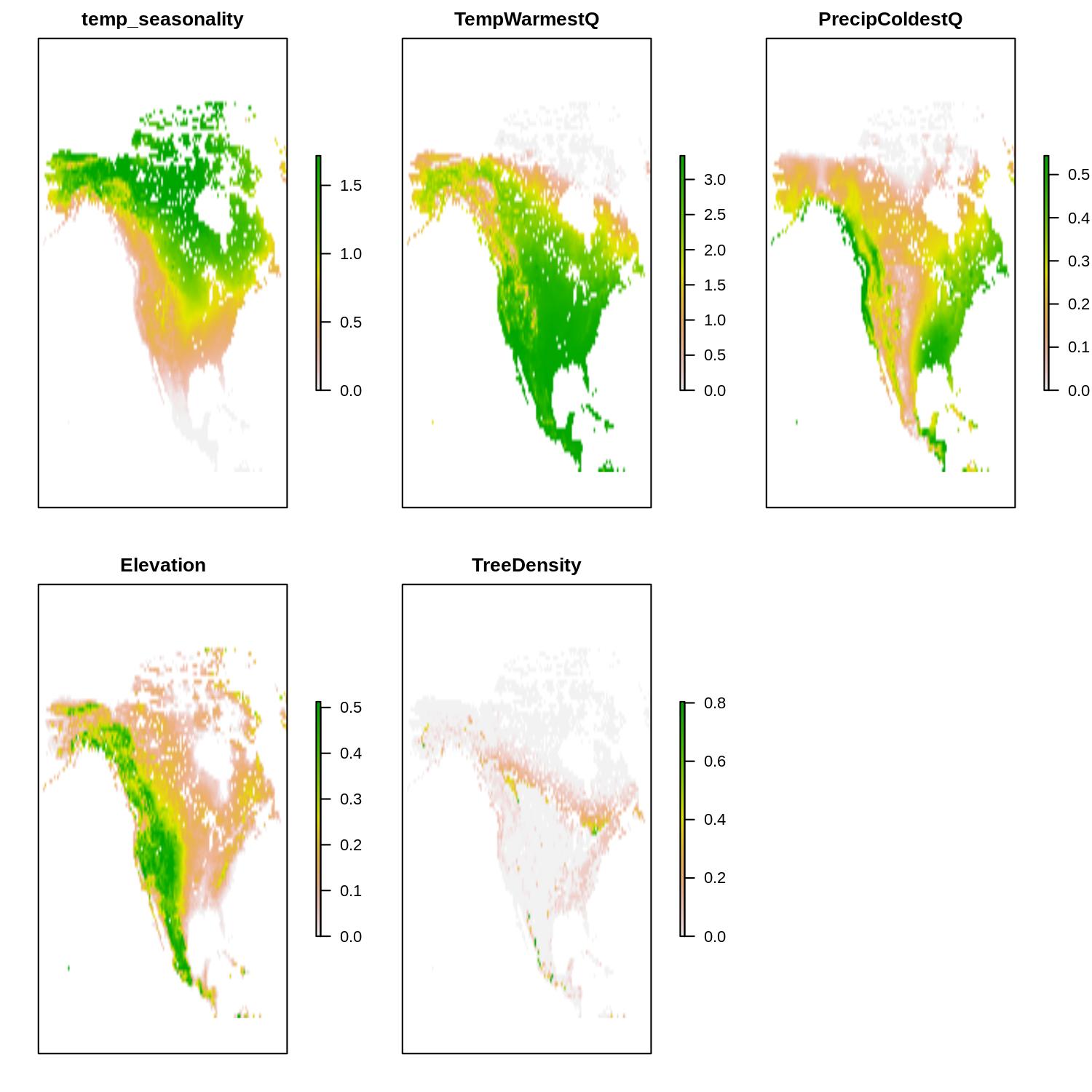


Figure S1.4: Individual environmental variables in the GDM transformed to biological space for W. Europe.


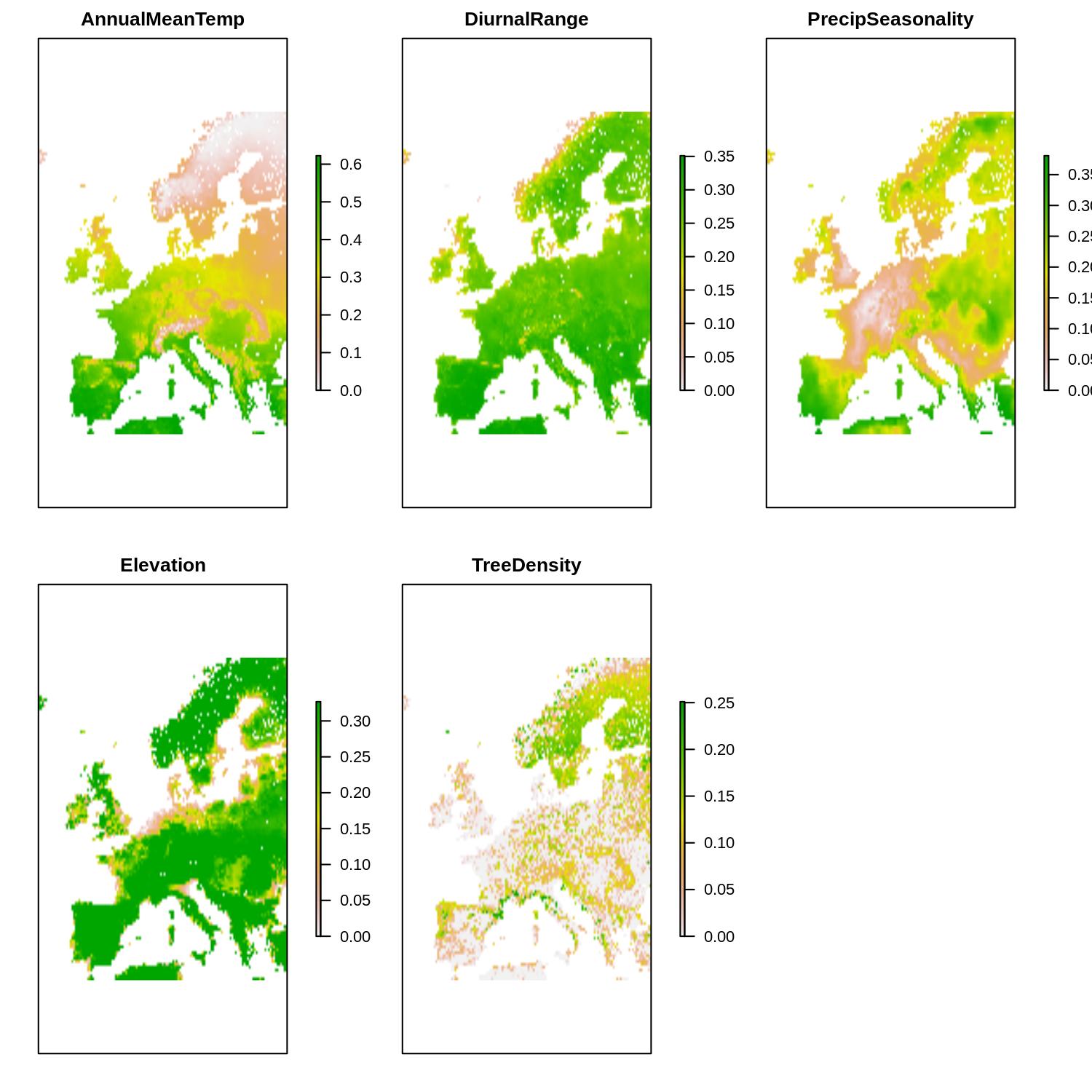


Figure S1.5: Fitted I-splines for significant environmental variables in the GDM run on Lepidoptera observations only; Lepidoptera being the most data-rich insect order, while also ecologically homogeneous relative to other species-rich insects. Y-axis shows the partial ecological distance, i.e. the magnitude of compositional change according to the environmental gradient.


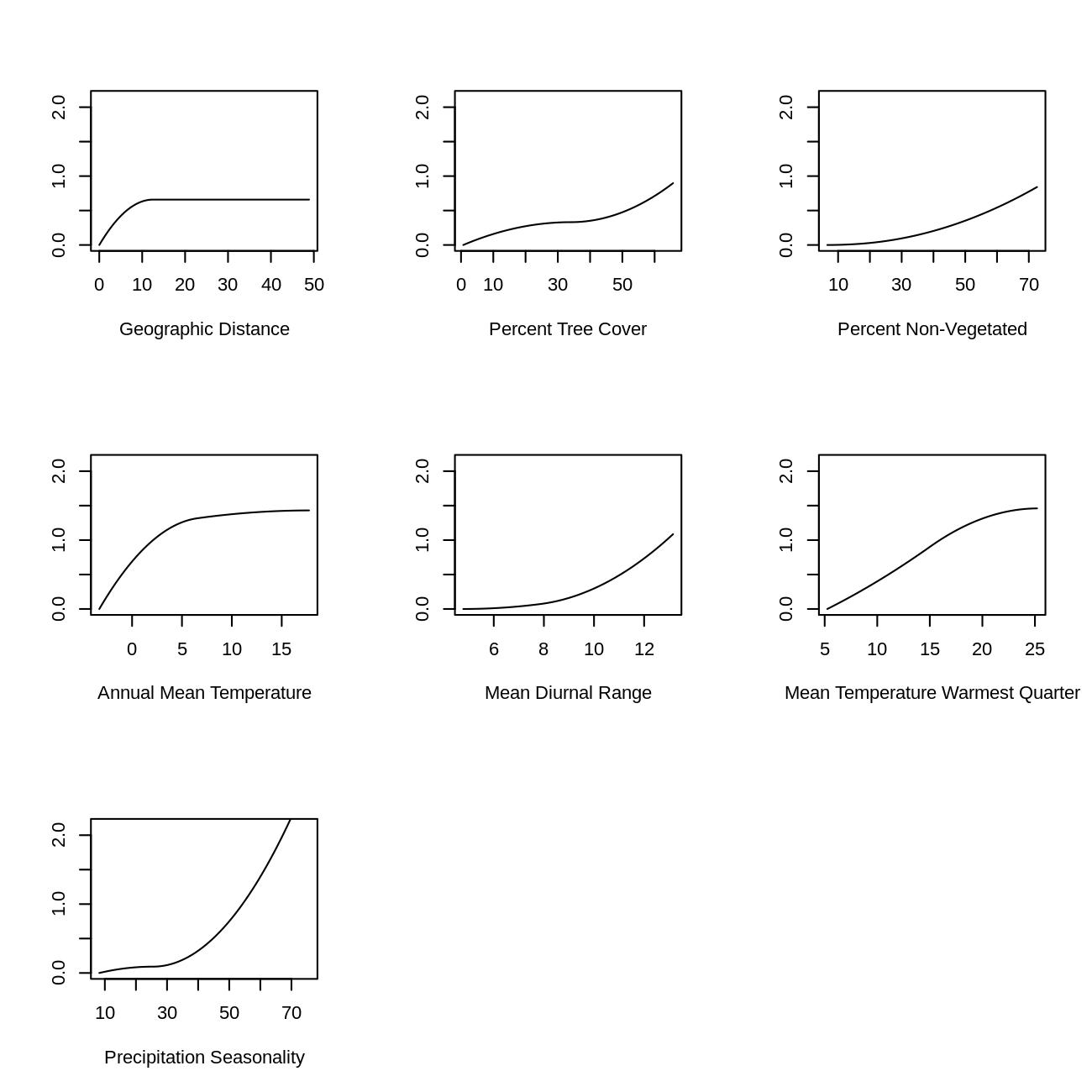

Supplement: Supplementary file 1 [file ECE3-9-13764-s001.docx]
